# An in vitro evaluation of the effect of sandblasting and laser surface treatment on the shear bond strength of a composite resin to the facial surface of primary anterior stainless steel crowns

**DOI:** 10.4317/jced.51876

**Published:** 2015-02-01

**Authors:** Nikhil Grover, Bhojraj Nandlal

**Affiliations:** 1 Reader, Department of Pedodontics & Preventive dentistry, School of Dental Sciences, Sharda University, Greater Noida, U.P; 2 Principal, Professor and Head, Department of Pedodontics and preventive dentistry, JSS Dental College, Mysore, India

## Abstract

Objectives: The present study was conducted to evaluate the optimal method of enhancing the bond strength of a composite resin to the facial surface of the primary anterior stainless steel crowns using various surface treatments namely Nd: YAG laser surface treatment, sandblasting , alloy primer application and no surface treatment. Study Design: The study sample consisted of 60 primary anterior stainless steel crowns (UnitekTM size R 4), with 15 samples randomly divided into the 4 study groups, embedded in acrylic blocks. The facial surface of these surface treated crowns was utilized as the bonding surface to which 2.5mm diameter composite resin cylinders were bonded for the evaluation of the shear bond strength. Shear bond strength measurements were made using a universal testing machine utilizing a shearing blade (jig).The mode of failure at composite-metal interface was determined using a Stereomicroscope at 10 X magnification. Results: The mean bond strength values obtained for surface treatment of Nd: YAG laser surface treated, Sandblasting ,Alloy Primer and No surface treatments were 17.01±.92 , 13.18 ± .73, 7.46 ± .70 and 7.33 ± .77 MPa respectively. The obtained bond strength values were subjected to a one way ANOVA and a Scheffe’s post-hoc comparison test. The results of the present study indicated that Laser surface treatment of the facial surface of the crowns enhanced the bond strength of the composite resin significantly compared to the other groups. Conclusions: Nd: YAG laser surface treatment produced an excellent surface roughness and obtained the highest shear bond strength values suggestive for recommendation as an optimal surface treatment to be used to enhance the resin-metal bond at the interface of the composite resin and the facial surface of primary anterior stainless steel crowns for the purpose of chairside veneering.

** Key words:**Nd: YAG laser treatment, Sandblasting, Primary anterior stainless steel crown, Chairside veneering, Enhancing Resin-Metal Bond, Early childhood caries, Shear bond strength (SBS).

## Introduction

Restorations for the primary anterior teeth have always been a challenge for the dentist for many decades (1). Carious involvement of the maxillary incisors not only compromises the integrity of the dentition but also creates an undesirable esthetic appearance (2).

 It has been a challenge to find a product that is biologically, mechanically, esthetically acceptable and offers a prolonged life expectancy in the mouth (3).

The various restorative modalities that have been used to treat primary anterior teeth are polycarbonate crowns, conventional stainless steel crowns, open-faced stainless steel crowns, strip crowns, commercially veneered crowns and composite shell crowns and recently the pedonatural crown and the current new entrant to this vast array preformed pediatric zirconia crowns (1,2,4).

 Additionally, there are some commercially available preformed crown forms made of co-polyester (PedoJackets, Space Maintainers Laboratory, and USA) but no literature reports could be found on these (4).

Primary anterior stainless steel crowns that have been veneered with a composite resin facing are a cost effective, aesthetically acceptable and durable restorative option for carious primary incisors. For the clinical longevity of this restoration satisfactory bond strength of the stainless steel crown facial surface to composite resin is of critical importance(1,5).

Advances in restorative materials and metal bonding procedures have made possible techniques that combine the advantages of stainless steel crowns with cosmetics of composites. Earlier studies have evaluated the effect of mechanical preparation; sandblasting and use of metal primers in enhancing the composite to metal bond (1,3,5).

To improve the mechanical bonding between metal and ceramic several methods have been introduced sandblasting, acid etching, application of bonding agents, laser sintering and laser etching among those laser etching is a surface treatment, which makes easier and also enables control of micro topography because of its depth of optical penetration depending on the material irradiated and provides more surface roughness and a stable surface morphology (6).

Other studies have evaluated the effect of laser surface preparation using an Nd: YAG, XeCl or Er, Cr: YSGG laser as a means to improve the metal-resin bond or improve the bond at the titanium-ceramic interface (7-11).This study was conducted to evaluate the optimal method of enhancing the bond strengths of anterior primary stainless steel crowns to a composite resin using surface preparation techniques sandblasting, alloy primer and the possibility of using laser surface treatment (Nd:YAG laser). The hypot-hesis was that the stainless steel crown surface treated with the Nd: YAG laser would influence the bond strength of a composite resin to these crowns by producing surface roughness to enhance the bond.

## Material and Methods

Statistical advice was sought and sample size calculated using data from a pilot in vitro experiment. The power of the sample was calculated using the G-Power 3.1.3 power analysis software (Franz Faul, Universitat Kiel, Germany). The minimum required sample for the one-way ANOVA and post-hoc test, with alpha of 0.05, was 15 samples in each group. The final study sample consisted of 60 primary anterior stainless steel crowns (UnitekTM size R 4) embedded in acrylic blocks. The four test groups had 15 samples each which underwent different surface treatments namely sandblasting, Nd: YAG laser surface treatment, metal (alloy) primer and no surface treatment (Control). Gluma composite material was used ( Table 1).

**Table 1:**
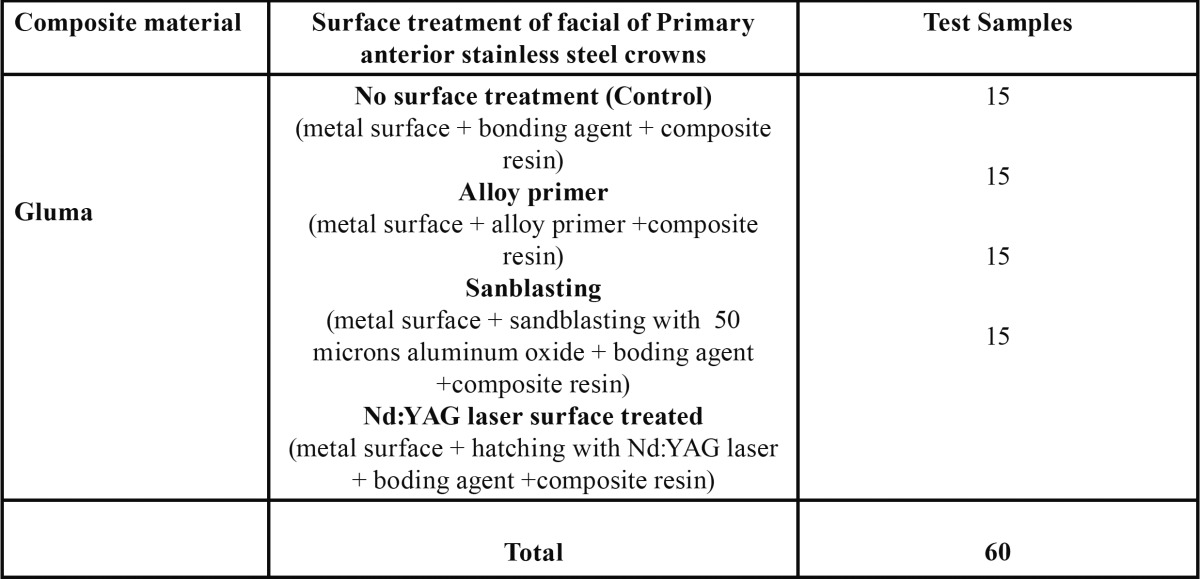
Distribution of samples according to the various surface treatments of Primary anterior stainless steel crowns (UnitekTM size R 4).

The mounting of the crowns was done using a square mould which was designed of specific dimensions with silicone duplicating material (BEGO, Germany). The position of the crowns in all the samples was standardized. The whole mould was filled with the pink acrylic using the same sprinkle method. The mould was then placed in a pressure pot to help in uniform polymerization and minimizing any porosity.

 The samples were then randomly divided into 4 groups of 15 samples each. Colour coding of the various groups was done to ensure identification of the test group’s laser surface treated: black; sandblasted group: red; alloy primer treated group: blue and no surface treatment group (Control): pink.

**Figure 1:**
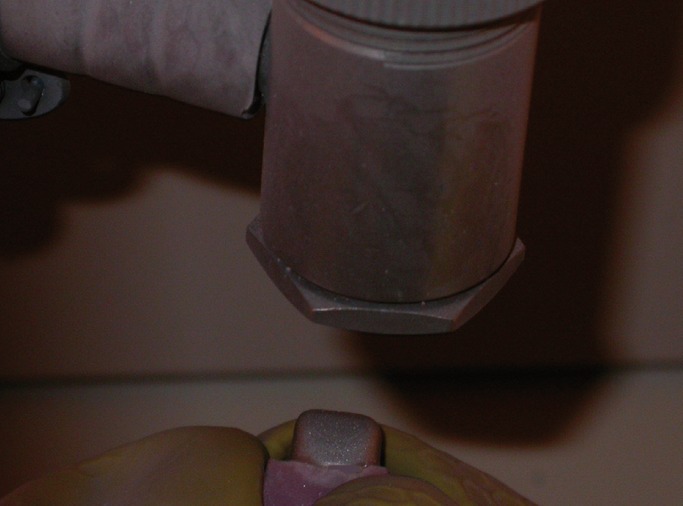
Sandblasting of the Primary Anterior Stainless Steel Crown.

The crowns sandblasted with a 50 microns aluminum oxide at a pressure of 75 psi for approximately 15 seconds resulting in the labial surface of the crowns to a dull frosty appearance (Fig. 1). The sandblasted crowns were bonded to the composite resins using the same procedure as described earlier but making sure that the bonding procedure was carried out within thirty minutes of sandblasting as strengths of sandblasted metals have been found to be affected adversely by a delay between sandblasting and bonding to composite (6).

The crowns were wiped with an alcohol swab followed by a dry towel. The metal bonding agent (Alloy Primer) was applied directly to the crowns labial surface with a brush for 15 seconds and then air dried for 5 seconds. The composite resin was then immediately bonded to the primary anterior stainless steel crowns facial surface using the same procedure as described earlier.

Laser of surface preparation the labial surface was carried out using LASER CHEVAL (Nd: YAG Laser; CF 11-75(60)). The CF 11- 75(60) is a high speed high precision class IV laser micromachining system that uses coherent light energy to produce a mark on the surface of metals (12). The Primary Anterior Stainless Steel crowns were placed on the work surface behind the screen which prevents the laser rays from affecting the eyes. Then a laser beam was focused on the facial surface of the primary anterior stainless steel crowns.

**Figure 2:**
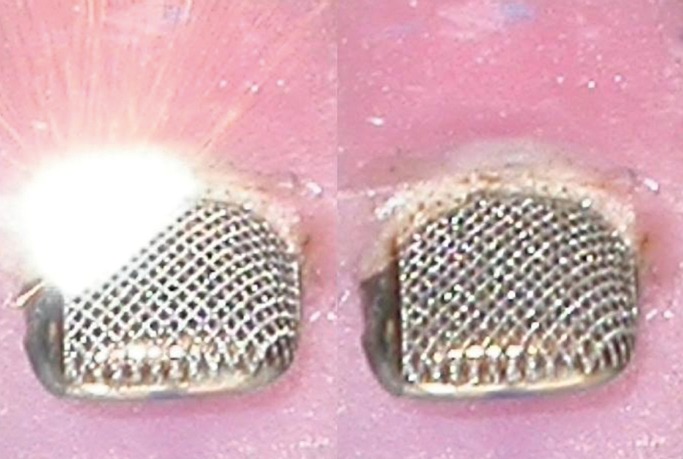
Laser surface treatment of the Primary Anterior Stainless Steel Crowns.

The parameters used for the Laser surface treatment of the crowns in the present study were 100% Power; Speed: 1000 mm/s; Frequency 8 KHz and a Pulse length of 3 microseconds. All the parameters were controlled using TRUVIEWTM or LENS job editor software. In addition using this software a Hatch pattern with 45 degrees and 135 degrees was created on the crown to enhance retention (Fig. 2).

To consistently place the composite material on the same location and of same amount on the anterior primary stainless steel crowns a template was constructed using a Copyplast sheet (code 172) in the Biostar (Scheu-Dental) using a positive pressure method so that a composite cylinder of 2.5mm × 3mm would be made for all the samples. The Copyplast material does not react with composite and is recommended by the manufacturers for use with composite resin.

The bonding agent given by the manufacturer (Gluma-Comfort bond) was applied on to the labial surface of the anterior primary stainless steel crowns as per the manufacture instructions. The composite resin (Gluma-Charisma) was taken on a clean plastic instrument and placed into the well of the Biostar template. The composite material was placed in the well in two increments of 1-1.5 mm each. The light cure gun was placed directly on top of the template well (cylinder) and photo cured for 20 seconds.

The cured samples were placed in distilled water and stored in an incubator at 37°C for 48 hours. The samples were thermocycled between 4°C and 55°C for 500 cycles with a dwell time in each thermal bath of 1 minute (13).

**Figure 3:**
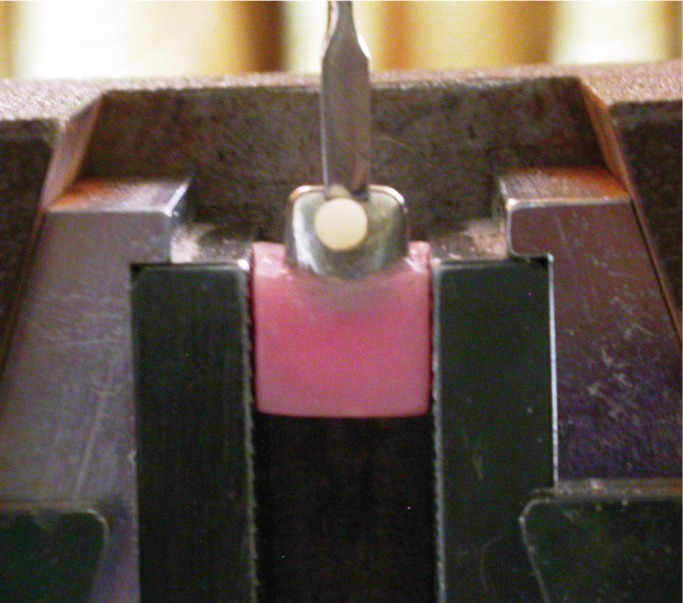
Specimen mounted on the Universal Testing Machine (Hounsfield U.K.).

Shear bond strength measurements were made using a universal testing machine (Hounsfield U.K. Model 50 KM with a capacity of 50 KN).The testing was done in a compression mode, in the lower jaw the sample was placed, in the upper jaw a shearing jig (0.5 m edge) was placed to shear the composite cylinders (2.5mm internal diameter and 3mm height) from the facial surface of the primary anterior stainless steel crowns (Fig. 3).

The edge of the shearing blade was kept 0.5 mm away from the surface of the crowns. The machine was operated at a cross head speed of 1.0 mm/min and the maximum values to debond the specimen were recorded in Newtons (N). The values so obtained were then converted into MPa using the known surface area of the composite cylinder.

The site of fracture between the composite cylinders and the facial surface of the surface treated primary anterior stainless steel crowns was determined using a Stereomicroscope at 10 X magnification. The advantage of using a stereomicroscope is that specimens needed no sectioning for viewing.

## Results

The mean bond strength values obtained for surface treatment of Laser surface treated, Sandblasting, Alloy Primer and No surface treatments were 17.01±.92 , 13.18 ± .73, 7.46 ± .70 and 7.33 ± .77 respectively ( Table 2).

**Table 2:**
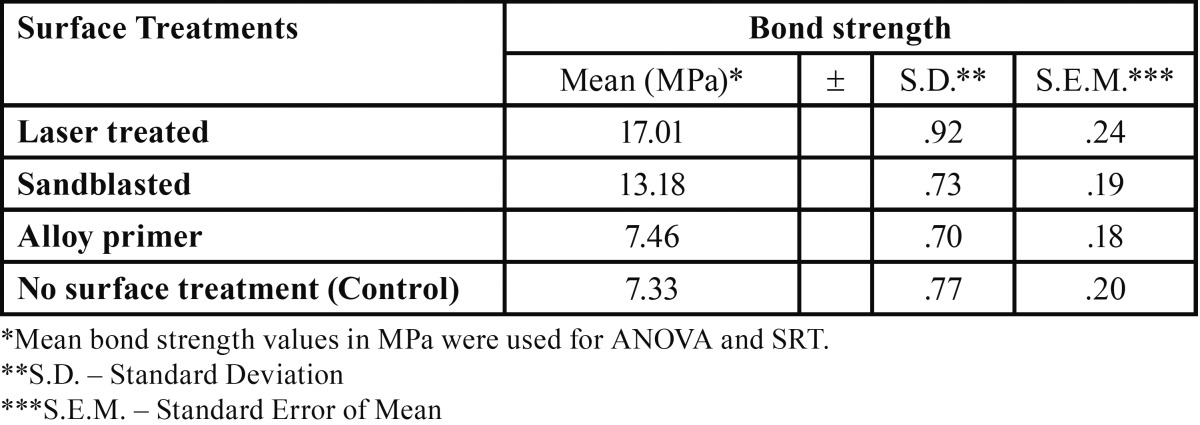
Mean Bond strength Values of Various surface treatments of Primary anterior stainless steel crowns.

The One way ANOVA made it clear that there was a significant difference in the mean bond strength values of different treatments. F value of 541.656 with 3 and 56 degrees of freedom is found to be highly significant (*P*<.001). The mean bond strength values for different treatments like laser treatment, sandblasting, alloy primer and control groups were 17.01, 13.18, 7.46 and 7.33 respectively.

The Scheffe’s post-hoc comparison test revealed that there was no significant difference in the mean values of control and Alloy primer treatments, which had the least bond strength, whereas laser surface treated group mean found to be significantly different from all other mean bond strength values having highest value. The mean bond strength value of sandblasting technique was also found to be different from others having second highest value ( Table 3).

**Table 3:**
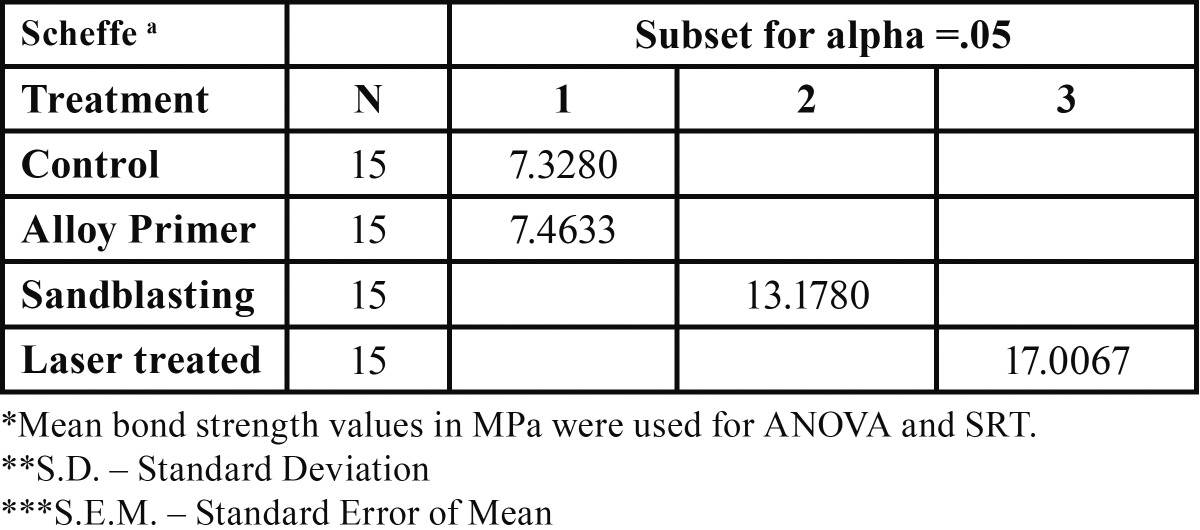
Scheffe’s Range Test of various surface treatments.

Fracture site distribution revealed that for the Nd: YAG laser surface treated group the fracture site distribution observed was: Adhesive Failure: 0 samples (0%); Cohesive Failure: 9 Samples (60%) and Combined (mixed) Failure: 6 Samples (40 %). For the sandblasting treated group the fracture site distribution observed was: Adhesive Failure: 2 samples (13.3%); Cohesive Failure: 2 Samples (13.3%) and Combined (mixed) Failure: 11 Samples (73.3 %). In the Alloy primer and No surface treatment surface treated group the fracture site distribution observed was: Adhesive Failure: 15 samples (100%).

## Discussion

Nursing bottle caries or baby bottle tooth decay is a common and serious condition affecting infants and preschool children; the prevalence of nursing bottle caries is predominant in the preschool population globally (14). The ideal full coronal restorations for a primary incisor should have the following characteristics: is tooth coloured and imperceptible; is durable enough to last in the mouth, with no additional treatment, until normal exfoliation time of the tooth; Must be adhesively attached to the prepared tooth with cement that will be biocompatible with pulp tissue; is easily and rapidly placed by the dentist; Should be placed in one treatment visit without need for laboratory fabrication of the crown (1-4).

An extensive systematic review of the dental literature in 2006 by Waggoner WF concerning the full coronal coverage of primary anterior teeth was performed, he found no clinical studies that could be identified that met all or even a majority of the criteria, indicating that there was little, good scientific support for any of the clinical techniques which clinicians have utilized for many years to restore primary anterior teeth (4).

Preformed stainless steel crowns have stood the test of time in pediatric dentistry as an outstanding restorative material for restoring primary teeth however the highly displeasing esthetic appearance of the crowns masks all the advantages to overcome this disadvantage various attempts have been made to convert this crown into a more esthetically pleasing one (1,2).

The Open faced stainless steel crown was an attempt to improve esthetics but had the disadvantages of being a time consuming procedure. The metal margins were visible and were operator sensitive as a variety of materials are used. Cutting the stainless steel metal inside the mouth was dangerous as particles might injure the patient. Hemorrhage could further compromise esthetics during placement of the resin (1,3,13).

Keeping in mind the disadvantages of all the other restorative modalities available, a chairside veneering technique was proposed which has the advantages of being durable and esthetically pleasing. The technique is substantially less expensive than the pre-veneered crowns. The technique retains the ability to crimp and adapt the crown to the tooth before veneering. Hemorrhage and saliva do not play a critical role in longevity of the restoration and the crowns can be sterilized (1,5).

The veneered primary anterior stainless steel crowns meet most of the demands of a biologically, mechanically and esthetically acceptable restorative material. For the success of the success of the veneered primary anterior stainless steel crowns the joint interface between the facial surface of the crown and the composite resin plays a critical role. The methods of increasing the bond between the crown and the composite resin would greatly enhance this weak joint thereby increasing its clinical success rate. Hence, this study was aimed to evaluate the optimal method of enhancing the bond strength of a composite resin to the facial surface of the primary anterior stainless steel crowns and if laser hatching using an Nd: YAG laser on facial surface of these crowns would have an effect on the bond at the resin-metal interface.

Various surface treatments have been employed to increase the bond strengths between the metal and resin. They include mechanical retention methods like undercuts and roughening of the metal surface, micro retention methods like sandblasting, electrolytic etching and tin-plating, laser surface treatment and chemical adhesion through the use of metal bonding agents (Metal Primers) (15-17).

Mechanical methods of roughening have inherent disadvantages that it might weaken the crown and a uniform roughening pattern might be difficult to achieve for all the samples. Further electrolytic etching has been found to be alloy specific, technique sensitive and requires the handling and storage of potentially harmful acids. Tinplating requires the use of specialized equipment’s, is technique sensitive and cannot be used intra-orally as the plating solution may cause injury if ingested or comes in contact with eye (17).

In the present study different surface treatments like laser surface treatment, sandblasting, and metal primer were included along with no surface treatment to evaluate the optimal method of enhancing the bond strength of a composite resin to the facial surface of primary anterior stainless steel crowns. Laser surface treatment in which a hatch pattern was created to mimic a meshwork on the facial surface of the primary anterior stainless steel crowns utilizing a Nd: YAG laser enhanced the bond strength between the facial surface of primary anterior stainless steel crowns and a composite resin most effectively.

Studies have shown that laser surface treatment produced an excellent surface roughness and achieved good shear bond strength values and aid in achieving a better bond strength between metals and ceramic (6).

Laser technology has been considered in almost all fields of dentistry. Some recent investigations, studied the effect of laser irradiation on the bond strength of resin restorative materials to ceramics. It has been reported that comparing with conventional sandblasting and acidetching techniques, there was no significant difference associated with the Er:YAG or Nd:YAG laser application to bond the resin cements to the dental porcelain, so treatment of porcelain surface with laser may be as effective as conventional methods (8).

In a study that evaluated the effect of laser etching using a Nd:YAG laser on shear bond strength between base metal alloys and ceramic, the results of the study indicated that the shear bond strength between ceramic bonded with Ni Cr alloys using the laser etching as surface treatment was significantly higher than the other groups they attributed this increase to the observation that the Nd:YAG laser etching increased surface roughness of the base metal alloys when compared to sandblasting, which would allow greater micro mechanical bonding which is in accordance with the present study (6).

A recent study compared the effects of XeCl laser etching of Ni–Cr alloy on bond strengths to composite resin and compared it with sandblasting procedures they concluded that laser pre-treatment of Ni–Cr alloy increased bond strength to composite resin compared with sandblasting, a similar result was seen in the present study albeit the laser used was Nd:YAG (7).

Madani, A *et al.* evaluated the effect of irradiation using a Nd:YAG laser on ceramic-covered alloy surface and hypothesized if it would improve the bond strength of resin to metal, and if different parameters of laser output may influence the strength of this bond, they concluded that shear bond strength was significantly higher in porcelain-covered laser treated samples, but the effect of power output of laser irradiation was not significant they further concluded that Nd:YAG laser surface treatment may improve the silica coating of alloy surface to achieve better resin–metal bond (8). Another study concluded that laser etching of titanium surfaces using an Nd: YAG laser was effective in improving bond strength with low-fusing porcelain, as compared to the acid-etching method (10).

Manufacturers of orthodontic devices have molar band products available commercially that have enhanced bond strength primarily due to surface modifications, such methods include laser surface treatment (18).

Roughening the metal surface improves the adhesive interfacial bond strength; this is primarily due to the increased surface area available for bonding and also due to mechanical interlocking feature. Similar mechanical retention represents the predominant mechanism in bracket bonding technique through a mesh base (9,18). It is important to be able to produce the roughened surface repeatedly and uniformly in order to ensure a high degree of reproducibility the process parameters must be tightly controlled which is possible using a laser (18). In the present study the Nd:YAG laser offered the same advantages as the hatch pattern could be reproduced and parameters controlled using the TRUVIEWTM or LENS job editor software.

Exposure of the test samples to cyclic thermal fluctuations to simulate one of the many factors in the oral environment has been common in many tracer penetration, marginal gap and bond strength laboratory tests. In the present study, thermo cycling of the samples at 4°C and 55°C for 500 cycles with a dwell time in each thermal bath of 1 minute was done to further stress the interface. [13] Van Meerbeek *et al.* have recently stated that the thermocycling strategy to accelerate bonding degradation must be performed using a number of cycles up to 100,000 to discriminate significant differences. They also highlighted that the ISO’s recommendations of thermocycling regimens (500 cycles) are of little use (19). It has also been stated that shear bond strengths results may be inferior, when extended thermocycling time is applied also according to ISO/Technical specification (TS) 11405, crosshead speed does not seem to influence bond strength values (6).

The use of Metal Primers on Primary Anterior stainless steel Crowns had lower bond strength than Laser surface treatment and Sandblasting with the composite material (Gluma). No surface treatment of the Primary Anterior stainless steel Crowns had lowers bond strength’s than Nd:YAG laser surface treatment, sandblasting and alloy primer groups.

Further, the fracture site distribution of the surface treatments revealed that laser surface treatment led to no adhesive fractures for all the samples tested thus recommending it as a technique for improving the bond between the composite veneer and the facial surface of the primary anterior stainless steel crowns. Sandblasting also enhanced the bond strength significantly although not as much as the laser treated group. It has been stated that shear bond strength in terms of nominal stress values is questionable due to the heterogeneous stress distribution and also due to the occurrence of cohesive failures both in the dental substrate and the resin composite. Defining categories for classification of failure modes of deboned specimens is a complicated task and in some instances, the limit between mixed and cohesive failure becomes merely subjective. Rather than an indication of strong bonding, cohesive failure is explained by the mechanics of the test and the brittleness of the materials involved (20).

Successful long term bonding requires proper technique and deep knowledge over dental materials as well as control over pre treatment techniques. The most important individual factor in order to achieve the highest possible shear bond strength is to choose a reliable bonding system and standardization of the surface treatments (6).

## Conclusions

Laser surface treatment obtained the highest bond strength suggestive for recommendation as optimal surface treatment method to be incorporated for the purpose of veneering Primary anterior stainless steel crowns. It can be recommended to the manufacturers of these crowns to provide crowns that are pre laser surface treated which will provide optimal bond strength between the composite veneer and the crown and also save valuable chair side time with a nominal increase in cost.
